# Supplementary material for: Genome-Wide Association Study Identified Candidate Genes for Alkalinity Tolerance in Rice
Source: Plants (Basel). 2023 Jun 3;12(11):2206. doi: 10.3390/plants12112206 (PMC10255560; doi:10.3390/plants12112206)
Supplement: Supplementary file 1 [file plants-12-02206-s001.zip › plants-2403389-supplementary.pdf]

Table S1. Phenotypic performance of rice genotypes in the control environment at the seedling stage.

**Trait[a]**	**Min**	**Max**	**Mean**	**Std_dev**	**RIL Pr > Fc[b]**	**Heritability**
AKT	1.0	2.0	1.2	0.43	0.22[ns]	0.92
SHL	25.88	70.37	45.47	7.66	0.37[ns]	0.86
RTL	11.54	80.78	26.99	5.20	0.29[ns]	0.71
RSR	0.21	2.10	0.61	0.17	0.16[ns]	0.83
Inv_FW	0.39	3.66	1.40	0.78	0.38[ns]	0.90
log_DW	-1.3	0.17	-0.23	0.28	0.28[ns]	0.79
SNC	120.15	976.80	518.36	151.98	0.32[ns]	0.81
SKC	665.86	1304.96	998.31	128.16	0.42[ns]	0.91
SNK	0.11	1.03	0.52	0.17	0.52[ns]	0.86

[a]AKT, alkalinity tolerance score; SHL, shoot length; RTL, root length; RSR, root-to-shoot ratio; inv_FW, inverse fresh weight; log_DW, log dry weight; SNC, shoot Na^+ concentration; SKC, shoot K^+ concentration; SNK, shoot Na^+: K^+ ratio.

[b]genotypic difference among lines.

[ns]non-significance differences between the means of genotypes

Fc – Analysis of Variance test

Table S2. Eigen value, variance, and cumulative variance (%) of principal components for nine morphological and physiological traits in the rice genotypes under alkaline stress.

Principal Components	Eigen values	Variance %	Cummulative Variance %
PC 1	3.43	38.16	38.16
PC 2	1.99	22.07	60.22
PC 3	1.68	18.63	78.85
PC 4	1.04	11.57	90.42
PC 5	0.7	7.75	98.18
PC 6	0.08	0.86	99.04
PC 7	0.04	0.44	99.48
PC 8	0.02	0.27	99.75
PC 9	0.02	0.25	100

PC, Principal components.

Table S3. Eigen vectors and eigen values of the principal components for nine morpho-physiological traits of rice genotypes evaluated for alkalinity tolerance at the seedling stage.

	PC 1	PC 2	PC 3	PC 4	PC 5	PC 6	PC 7	PC 8	PC 9
AKT	-0.932	0.019	-0.25	0.06	-0.141	0.203	0.015	-0.049	-0.031
SHL	0.22	-0.595	-0.517	-0.561	-0.084	-0.003	-0.006	-0.054	0.07
RTL	-0.257	0.359	0.293	-0.845	-0.02	0.009	0.01	0.04	-0.05
RSR	-0.329	0.711	0.608	-0.043	0.048	-0.004	-0.004	-0.066	0.084
Inv_FW	0.902	-0.122	0.322	-0.032	0.163	0.19	-0.015	0.051	0.041
log_DW	0.929	-0.069	0.267	-0.045	0.152	0.006	0.035	-0.099	-0.072
SNC	-0.635	-0.453	0.17	-0.049	0.591	-0.009	-0.098	-0.012	-0.013
SKC	0.043	0.545	-0.655	-0.028	0.512	0.007	0.09	0.01	0.013
SNK	-0.491	-0.689	0.507	0.022	0.079	-0.015	0.141	0.014	0.018

PC, Principal components; AKT, alkalinity tolerance score; SHL, shoot length; RTL, root length; RSR, root-to-shoot ratio; inv_FW, inverse fresh weight; log_DW, log dry weight; SNC, shoot Na^+ concentration; SKC, shoot K^+ concentration; SNK, shoot Na^+:K^+ ratio.

Table S4. Mean value of each group identified by cluster analysis for nine morphological and physiological traits in the rice genotypes under alkaline stress at the seedling stage.

Group[a]	AKT	SHL	RTL	RSR	log_DW	Inv_FW	SNC	SKC	SNK
1 (HS)	8.11	36.30	16.43	0.46	0.06	-2.09	2055.61	601.63	3.81
2 (T)	3.61	38.32	15.85	0.43	0.20	-1.29	1625.55	629.25	2.72
3 (HT)	1.84	38.86	15.94	0.42	0.24	-0.90	1487.09	635.27	2.49
4 (MT)	5.67	38.07	16.66	0.45	0.12	-1.71	1792.92	709.92	2.59
5 (S)	7	33.11	18.73	0.71	0.07	-1.97	1956.05	730.70	2.68

[a]Five different groups identified by cluster analysis: HS, highly susceptible; T, tolerant; HT, highly tolerant; MT, moderately tolerant; S, susceptible. AKT, alkalinity tolerance score; SHL, shoot length; RTL, root length; RSR, root-to-shoot ratio; inv_FW, inverse fresh weight; log_DW, log dry weight; SNC, shoot Na^+ concentration; SKC, shoot K^+ concentration; SNK, shoot Na^+:K^+ ratio.

Table S5. List of rice genotypes, source, subspecies category, and the sub-group classification by the software 'STRUCTURE'.

Sl. No.	Lines	Source	Subspecies	Subgroup (SG)
1	Hasawi	Saudi Arabia	*indica*	SG4
2	PSBRC-50	Philippines	*indica*	SG3
3	Roy J	Louisiana	*japonica*	SG1
4	CL111	Louisiana	*japonica*	SG1
5	Djogolan	Burkina Faso	*indica*	SG5
6	Saturn	Louisiana	*japonica*	SG1
7	Caloro	California	*indica*	SG2
8	LAH10	Louisiana	*japonica*	SG5
9	Cheriviruppu	India	*indica*	SG5
10	Pinkaeo	Thailand	*indica*	SG4
11	CL142	Louisiana	*japonica*	SG1
12	CL131	Louisiana	*japonica*	SG1
13	LAH10	Louisiana	*japonica*	SG3
14	Della	Louisiana	*japonica*	SG1
15	JN100	Louisiana	*japonica*	SG2
16	Century Rogue	Texas	*japonica*	SG1
17	Pokkali	Srilanka	*indica*	SG5
18	Dular	Bangladesh	*indica*	SG2
19	Mermentau	Louisiana	*japonica*	SG1
20	Cypress	Louisiana	*japonica*	SG1
21	Evangeline	Louisiana	*japonica*	SG2
22	Trenasse	Louisiana	*japonica*	SG1
23	Vegold	Arkansas	*japonica*	SG1
24	Pecos	Texas	*japonica*	SG2
25	Nona Bokra	India	*japonica*	SG5
26	Moroberekan	Guinea	*japonica*	SG2
27	Wells	Arkansas	*japonica*	SG1
28	JN349	Louisiana	*japonica*	SG2
29	Pirogue	Louisiana	*japonica*	SG2
30	ChN1264	Louisiana	*japonica*	SG1
31	Gold Zenith	Arkansas	*japonica*	SG2
32	Skybonnet	Texas	*japonica*	SG1
33	LA0802140	Louisiana	*japonica*	SG1
34	Nipponbare	Japan	*japonica*	SG2
35	Jupiter	Louisiana	*japonica*	SG2
36	LA0702085	Louisiana	*japonica*	SG1
37	Rexona	Louisiana	*japonica*	SG5
38	Toro-2	Louisiana	*japonica*	SG1
39	Belle Patna	Texas	*japonica*	SG1
40	Tebonnet	Arkansas	*japonica*	SG1
41	FL478	Philippines	*indica*	SG3
42	Geumgangbyeo	South Korea	*indica*	SG3
43	Neptune	Louisiana	*japonica*	SG2
44	CL261	Louisiana	*japonica*	SG2
45	Nira	Louisiana	*japonica*	SG1

46	Mercury	Louisiana	*japonica*	SG2
47	BHA1115	Arkansas	*indica*	SG4
48	Vista	Louisiana	*japonica*	SG2
49	FL318	Philippines	*Indica*	SG5
50	IR 29	Philippines	*indica*	SG2
51	Caffey	Louisiana	*japonica*	SG2
52	N22	India	*indica*	SG4
53	Magnolia	Louisiana	*japonica*	SG2
54	Lacassine	Louisiana	*japonica*	SG1
55	CLPK873	Louisiana	*japonica*	SG3
56	TCCP	Philippines	*indica*	SG4
57	Cocodrie	Louisiana	*japonica*	SG5
58	Templeton	Arkansas	*japonica*	SG1
59	Lacrosse	Louisiana	*japonica*	SG1
60	Dellrose	Louisiana	*japonica*	SG3
61	Nova 66	Arkansas	*japonica*	SG2
62	Lotus	Texas	*japonica*	SG1
63	IRRI147	Philippines	*indica*	SG2
64	R609	Louisiana	*indica*	SG3
65	Taggert	Arkansas	*japonica*	SG5
66	Agami	Egypt	*indica*	SG3
67	Sunbonnet	Louisiana	*japonica*	SG1
68	Lafitte	Louisiana	*japonica*	SG3
69	Bluebelle	Texas	*japonica*	SG1
70	Neches	Texas	*japonica*	SG2
71	Epagri	Brazil	*indica*	SG3
72	Cheniere	Louisiana	*japonica*	SG1
73	Jazzman-2	Louisiana	*japonica*	SG3
74	Arang	Indonesia	*indica*	SG3
75	Ecrevisse	Louisiana	*japonica*	SG1
76	Dellmati	Louisiana	*japonica*	SG5
77	Smooth Zenith	Texas	*japonica*	SG2
78	Carolina Gold	Texas	*japonica*	SG1
79	Damodar	India	*indica*	SG5
80	Bengal	Louisiana	*japonica*	SG2
81	Jes	Arkansas	*indica*	SG5
82	Kalia	Bangladesh	*indica*	SG2
83	Toro	Louisiana	*japonica*	SG3
84	Della 2	Louisiana	*japonica*	SG3
85	Century Patna	Texas	*japonica*	SG1
86	MS-1995-15	Mississippi	*japonica*	SG2
87	Chengri	Bangladesh	*indica*	SG2
88	CL152	Louisiana	*japonica*	SG5
89	CL162	Louisiana	*japonica*	SG3
90	SLO16	India	*japonica*	SG2
91	Nato	Louisiana	*japonica*	SG1
92	Rexark	Arkansas	*japonica*	SG3
93	MS-1996-9	Mississippi	*japonica*	SG1
94	Glutinous Selection	Texas	*japonica*	SG3

95	Saturn Rogue	Arkansas	*japonica*	SG3
96	Azucena	Philippines	*indica*	SG3
97	V20B	Philippines	*indica*	SG2
98	Langmanbi	India	*indica*	SG4
99	Milagrosa	Philippines	*indica*	SG5
100	Zhenshan 97	China	*indica*	SG2
101	CSR11	India	*indica*	Not Classified
102	R 50	Philippines	*indica*	SG5
103	Mars	Arkansas	*japonica*	SG5
104	Vandana	India	*indica*	SG3
105	Gu Ze	Japan	*japonica*	SG3
106	Ning Yang Keng	Taiwan	*indica*	SG3
107	IR 50	Philippines	*indica*	SG5
108	Kasalath	India	*indica*	SG5
109	Stormproof	Arkansas	*japonica*	SG5
110	R-54	Missouri	*japonica*	SG3
111	Starbonnet	Arkansas	*japonica*	SG2
112	Sarioo50	Arkansas	*japonica*	SG1
113	B573-A4-20-6	Texas	*japonica*	SG1
114	IR 8	Philippines	*indica*	SG3
115	M202	California	*japonica*	SG1
116	Zenith	Arkansas	*japonica*	SG5
117	R-27	Missouri	*japonica*	SG3
118	Gold Nato	Arkansas	*japonica*	SG2
119	Panidhan II	India	*indica*	SG5
120	Naylamp	Peru	*indica*	SG5
121	Azaurel	Venezuela	*indica*	SG4
122	IR 64	Philippines	*indica*	SG2
123	Koshihikari	Japan	*japonica*	SG3
124	Arkansas Fortuna	Arkansas	*japonica*	SG3
125	Melrose	Texas	*japonica*	SG3
126	Texmont	Texas	*japonica*	SG2
127	Kranti	India	*indica*	SG4
128	TP 49	Texas	*japonica*	SG2
129	Jinheung	South Korea	*japonica*	SG1
130	Teqing	China	*indica*	SG3
131	Taichung 65	Taiwan	*japonica*	SG3
132	Arkrose	Arkansas	*japonica*	SG1
133	Dixiebelle	Texas	*japonica*	SG5
134	Millie	Arkansas	*japonica*	SG5
135	Kirak	India	*indica*	SG2
136	Daido	Taiwan	*indica*	SG2
137	Nerretto	Italy	*japonica*	SG1
138	Lemont	Texas	*japonica*	SG5
139	Quilloa 66304	China	*indica*	SG2
140	PSRR-1	Louisiana	*indica*	SG2
141	Kokubelle	California	*japonica*	SG2
142	Dellmont	Texas	*japonica*	SG2
143	Chambal	India	*indica*	Not Classified

144	Chung yuen	China	*indica*	Not Classified
145	Bala	India	*indica*	Not Classified
146	Co39	Philippines	*indica*	Not Classified
147	IRGC1244	Philippines	*indica*	SG2
148	Newrex	Texas	*japonica*	SG5
149	Bluebonnet	Texas	*japonica*	SG5
150	RD	Texas	*japonica*	SG2
151	Kanchan	India	*indica*	Not Classified
152	San Tou Thou	China	*indica*	Not Classified
153	Pratao Tipo Guedes	Philippines	*indica*	Not Classified
154	Swarna	India	*indica*	SG5
155	IRGC32567	Philippines	*indica*	SG5
156	W149	Louisiana	*japonica*	SG2
157	Orion	Arkansas	*japonica*	SG5
158	IR4432-52-6-4	Philippines	*indica*	SG2
159	Perum karuppan	Srilanka	*indica*	Not Classified
160	Dholamon 560	Bangladesh	*indica*	SG2
161	Kitaake	Japan	*japonica*	SG5
162	Taipe 309	Japan	*japonica*	Not Classified
163	Hill Long Grain	Texas	*japonica*	SG2
164	Adair	Arkansas	*japonica*	SG2
165	Bharathy	India	*indica*	Not Classified
166	H4	Louisiana	*japonica*	SG1
167	IARI 5823	India	*indica*	SG5
168	Hayamasari	Japan	*japonica*	Not Classified
169	Early Prolific	Arkansas	*japonica*	SG3
170	Brazos	Texas	*japonica*	SG5
171	LaGrue	Arkansas	*japonica*	SG2
172	M-204	California	*japonica*	SG2
173	Fatehpur 3	Pakistan	*indica*	SG2
174	Delitus	Louisiana	*japonica*	SG1
175	Italica Livorno	Italy	*japonica*	SG2
176	Hill medium	Texas	*japonica*	SG3
177	Prelude	Arkansas	*japonica*	SG2
178	Jackson	Texas	*japonica*	SG2
179	WC10380	Philippines	*indica*	Not Classified
180	CT-329	India	*indica*	SG2
181	KN-1-B-361-1-8-67	Indonesia	*indica*	SG4

Table S6. List of primers used in the gene expression profiling by qRT-PCR.

Gene	Forward Primer	Reverse Primer
LOC_Os04g50090	CGCCAGATGAACGAGTACCT	TTCACGAAATCAATGGCGCC
LOC_Os08g23440	GCAATTACGATTGGACGCCC	GACTGCCATACACCTGCCAT
LOC_Os09g32972	TACCTTAGCCCCGACCTCAA	CGCTTGATGTGCTGTTCCAC
LOC_Os10g35230	AGCTCGGCTGCATACCAAAT	GGGCAGTCACCTCCATCATC
LOC_Os03g25480	TCAATGATCGGCAAAGGGCT	CCACCCCTGGATCAAGCATT
LOC_Os08g25390	CCCTGGTACCATGATCTGCC	CACCAGCCATTCCAGTTCCT
LOC_Os09g38340	CGCAGAATCCCACCAATCCA	CTCCGCATCTGGATTTGGGT
LOC_Os04g58160	TCTGCTTCATCACCTGCTGG	ATCACCAGCGTGCTGTACTT

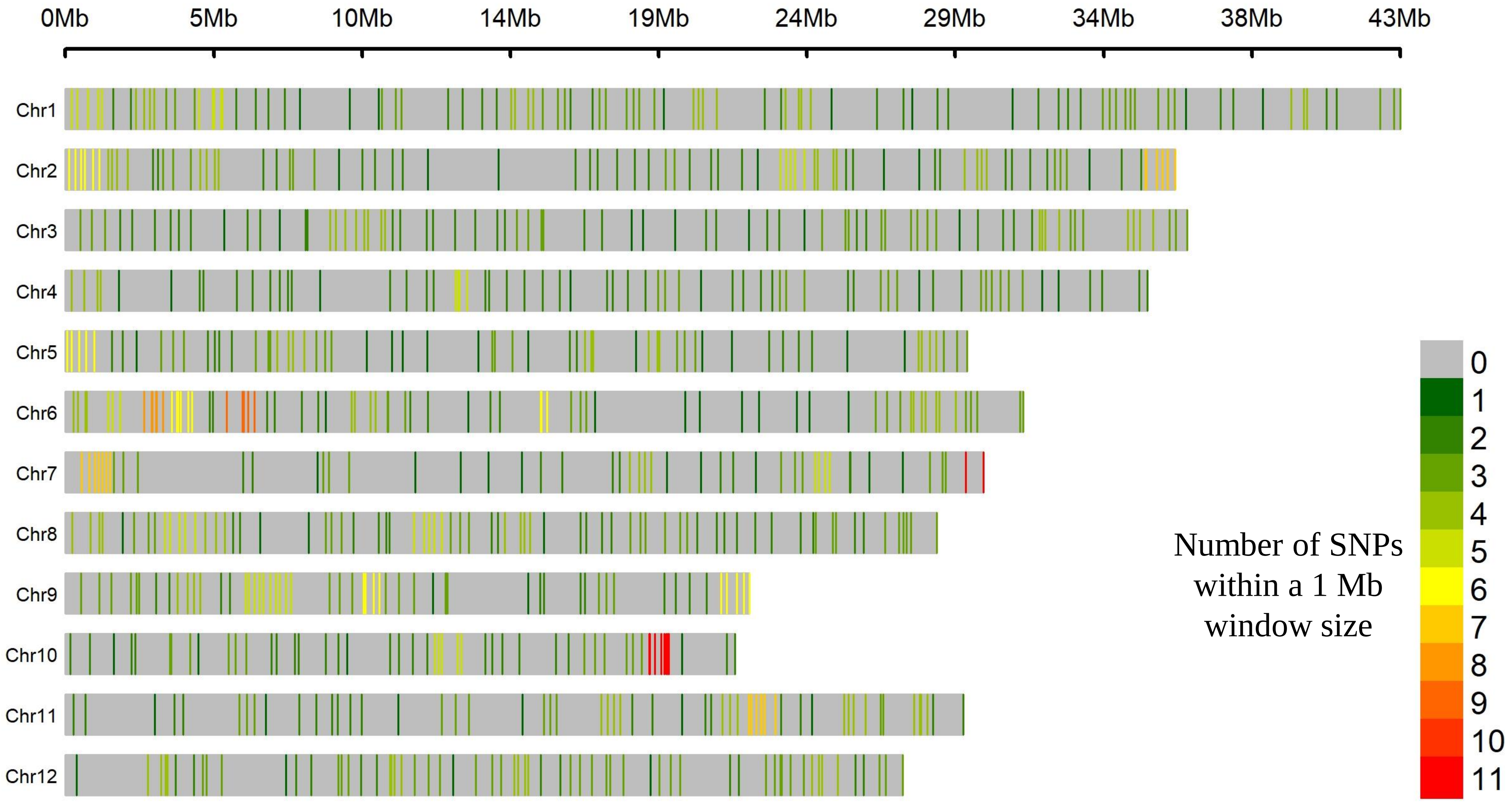

Figure S1. Linkage map of rice showing the number of SNPs within a 1Mb window size on each chromosome.
